# Peripheral miRNA profiling identifies therapy-specific biological trajectories in the treatment of cognitive dysfunction in depression: an exploratory mechanistic study

**DOI:** 10.1038/s41598-026-55039-1

**Published:** 2026-05-29

**Authors:** Lluis Miquel-Rio, Muriel Vicent-Gil, Judith Jericó-Escolar, Júlia Carrasco-Hernández, Miriam Jubero, Verónica Paz, Leonor Gawron, Esther Ruiz-Bronchal, Javi Vera, Caterina del Mar Bonnín, Dolors Puigdemont, Carlo Alemany, Narcís Cardoner, Javier de Diego-Adeliño, Analia Bortolozzi, Maria J. Portella

**Affiliations:** 1 Institute of Biomedical Research of Barcelona (IIBB), Spanish National Research Council (CSIC), Barcelona, 08036 Spain; 2 Systems Neuropharmacology Research Group, August Pi i Sunyer Biomedical Research Institute (IDIBAPS), Barcelona, 08036 Spain; 3 Biomedical Research Networking Center for Mental Health (CIBERSAM), Institute of Health Carlos III (ISCIII), Madrid, 28029 Spain; 4 Sant Pau Mental Health Group, Institut de Recerca Sant Pau (IR SANT PAU), Sant Quintí 77-79, Barcelona, 08041 Spain; 5 Hospital de la Sant Creu i Sant Pau, Sant Antoni Maria Claret 167, Barcelona, 08025 Spain; 6 Department of Psychiatry and Legal Medicine, Universitat Autònoma de Barcelona (UAB), Bellaterra, 08193 Spain; 7 Faculty of Medicine and Health Sciences, University of Barcelona (UB), Barcelona, 08036 Spain; 8 Addictions Unit, Psychiatry and Psychology Service, ICN, Hospital Clinic Barcelona, Health and Addictions Research Group, IDIBAPS, Barcelona, 08036 Spain

**Keywords:** Major depressive disorder, Cognitive dysfunction, Cognitive remediation, microRNA, Biomarker, Neuroplasticity, Biomarkers, Diseases, Neuroscience

## Abstract

Cognitive dysfunction in major depressive disorder (MDD) often persists despite standard treatment. Integral cognitive remediation (INCREM) shows clinical efficacy, yet its molecular drivers remain poorly understood compared to supportive interventions like psychoeducation (PSYCHOED). In this exploratory study, we investigated whether circulating microRNAs (miRNAs) reflect the distinct biological trajectories of these interventions. We conducted a longitudinal analysis of 38 candidate miRNAs (previously validated in postmortem brain tissue of MDD individuals) in plasma from 22 patients with MDD participating in a randomized clinical trial who were assigned to 12 weeks of INCREM or PSYCHOED. Using bioinformatic pathway analyses (KEGG/GO), we identified non-overlapping miRNA profiles associated with each treatment. The INCREM response was characterized by a specific seven-miRNA signature (let-7b-3p, miR-100-5p, miR-129-5p, miR-135a-5p, miR-151a-5p, miR-4516, and miR-451a) targeting gene networks regulated in neuroplasticity, axon guidance, and synaptic transmission. These molecular changes mirrored significant objective cognitive improvements. Conversely, PSYCHOED induced a distinct profile (miR-126-5p and miR-195-5p) related to systemic cellular homeostasis and Wnt signaling, without objective cognitive gains. Our findings suggest that psychological therapies act as specific biological stimuli with distinct molecular targets. These circulating miRNA signatures provide preliminary evidence of neuroplasticity-related pathways as key drivers of cognitive recovery, offering potential blood-based biomarkers for precision psychiatry in MDD.

**Supplementary Information:**

The online version contains supplementary material available at 10.1038/s41598-026-55039-1.

## Introduction

Major depressive disorder (MDD) is a prevalent and recurring mental illness, affecting one in five adults during their lifetime and representing a leading contributor to disability worldwide^1^. Although mood symptoms are central to the diagnosis, depressive disorder is also characterized by a wide array of cognitive symptoms, including difficulties in attention, memory, processing speed, and executive function^2–4^. These cognitive deficits are not merely a secondary consequence of the depressive mood, they are a core component of the illness^5^. Indeed, they play a key role in the psychosocial impairment experienced by people with MDD, hindering their performance at work and in education, and disrupting their social interactions^3,6^. Consequently, cognitive symptoms that remain untreated are associated with a lower quality of life and an increased risk of recurrent depressive episodes^7,8^.

Although the precise biological origins of MDD remain complex, the impaired neuroplasticity hypothesis provides a compelling explanatory framework^9–12^. Neuroplasticity ‒the neural capacity to reorganize structurally and functionally in response to stimuli‒ is fundamental for adaptation^13^. In MDD, this process becomes maladaptive, characterized by compromised synaptic strength, dendritic atrophy, deficiencies in neurotrophic factors, glial dysfunction, and altered functional connectivity^14–18^. Current first-line treatments, including antidepressants (selective serotonin reuptake inhibitors - SSRI), cognitive behavioral therapy (CBT), and electroconvulsive therapy (ECT), are believed to exert their clinical benefits by targeting and reversing these plasticity deficits in vulnerable brain regions, such as the dorsolateral prefrontal cortex (dlPFC)^19–24^. However, monitoring the molecular changes directly in the living human brain remains a significant challenge, underscoring the need for accessible peripheral biomarkers.

MicroRNAs (miRNAs), a class of small non-coding RNAs, have emerged as powerful candidates for this purpose due to their unique ability to control gene regulatory networks^25,26^. By interacting with messenger RNA (mRNA) transcripts, miRNAs orchestrate broad regulatory outcomes; a single miRNA can target hundreds of mRNA transcripts, while conversely, multiple miRNAs can converge to regulate a single mRNA transcript, acting as a true master switch for biological processes^27,28^. Indeed, there is compelling evidence linking changes in the regulation of miRNAs and their mRNA targets to the onset of MDD and antidepressant efficacy^29–34^. Notably, miRNAs can cross the blood-brain barrier and remain stable in the circulation, making them valuable candidates for liquid biopsies that could reflect pathophysiological states of the central nervous system (CNS)^35–37^. Evidence shows comparable dysregulation of miRNAs in the blood and brain tissue of patients with neuropsychiatric diseases, including MDD^38–41^.

Despite the significant impact of cognitive symptoms in MDD, therapeutic options remain an area of ongoing research. While pharmacological strategies have shown limited success, functional remediation programs, specifically integral cognitive remediation (INCREM), have been shown to improve the cognitive abilities and daily functioning of affected individuals^42^. However, the molecular mechanisms driving these benefits, and the way in which they differ from those of standard supportive care still need to be clarified. This study aims to investigate whether circulating miRNAs are associated with cognitive responses to INCREM and psychoeducation (PSYCHOED) in patients with MDD. A panel of 38 miRNAs, selected based on their prior dysregulation in post-mortem dlPFC samples from patients with MDD^43,44^, was analyzed to determine their potential as blood-based predictive biomarkers. Our findings reveal new mechanistic insights, showing that INCREM and PSYCHOED are related to different molecular pathways.

## Methods

### Study design and participants

This study was conducted as an exploratory translational analysis within a larger randomized controlled trial designed to evaluate the functional and cognitive outcomes of the INCREM program. The protocol was approved by the Research Ethics Board of the Hospital de la Santa Creu i Sant Pau and adhered to the Declaration of Helsinki and Good Clinical Practice guidelines, in full compliance with current data protection regulations. All participants provided written informed consent. The study is registered at ClinicalTrials.gov (30/05/2025, registration number NCT07014683) and the full protocol has been previously published^45^.

The study included outpatients diagnosed with MDD according to DSM-5 criteria. Inclusion criteria were: (i) age between 18 and 60 years; (ii) partial response to pharmacological treatment, defined as a 17-item Hamilton Depression Rating Scale (HDRS-17) score ≤ 18^46,47^, where no changes in antidepressant medication were permitted during the month prior to the study and throughout the study period; (iii) presence of objective cognitive deficits (screening for cognitive impairment in psychiatry [SCIP] ≤ 80)^48^ and/or subjective cognitive complaints (perceived deficit questionnaire [PDQ] > 20)^49^; and (iv) moderate psychosocial dysfunction (functioning assessment short test [FAST] ≥ 12)^50^. Exclusion criteria included neurological disorders, any medical condition that may affect neuropsychological performance, or comorbid psychiatric disorders, as detailed previously^45^. For this exploratory molecular sub-study, a total of 22 patients with complete paired plasma samples (pre- and post-intervention) were analyzed.

### Interventions and sample collection

The study followed a prospective, randomized, active-controlled design comparing two intervention arms: (1) INCREM, focusing on neurocognitive training and strategy acquisition; and (2) PSYCHOED, serving as an active control condition focused on illness management. Full intervention details are described in Vicent-Gil et al.^45^. At baseline (T_0_), participants underwent a comprehensive clinical, neuropsychological, and psychosocial functional assessment. Peripheral blood samples were collected under standardized conditions, e.g., between 8:00 and 10:00 AM after an overnight fast to minimize circadian variability in circulating biomarkers. Following baseline assessment, participants were randomly assigned to the INCREM or PSYCHOED arm using block randomization with balance allocation, based on a computer-generated sequence prepared by an independent statistician. Both interventions were delivered concurrently through structured weekly online group sessions for 12 weeks. Immediately post-intervention (T_1_), clinical and cognitive outcome measures were re-administered, and a second paired blood sample was obtained. A follow-up clinical and cognitive assessment (T_2_) was conducted six months post-intervention to evaluate the maintenance of therapeutic effects.

### Clinical and neuropsychological outcome measures

Sociodemographic and clinical data were collected using a semi-structured interview and standardized scales. These included: age; sex; years of schooling; estimated IQ (assessed using vocabulary subtest of the Wechsler Adult Intelligence Scale, 4th edition [WAIS-IV])^51^; age at illness onset; number of depressive episodes; depressive symptomatology (HDRS-17 item version)^46,47^; and months of clinical stability and antidepressant medication trials using the Antidepressant Treatment History Form (ATHF)^52^.

The primary outcomes for this translational analysis include: (1) psychosocial functioning measured using the Functioning Assessment Short Test (FAST)^50^, which evaluates impairment across six domains (autonomy, occupational performance, cognitive function, interpersonal relationships, financial management, and leisure activities). Scores ≥ 12 indicate moderate functional impairment. (2) Objective cognitive performance, measured using the Screening for Cognitive Impairment in Psychiatry (SCIP) tool^48^, which comprises five subtests targeting immediate and verbal learning and memory, working memory, verbal fluency, and processing speed. Scores < 80 indicative objective cognitive deficits. (3) Subjective cognitive functioning, evaluated using the 20-item Perceived Deficit Questionnaire (PDQ-20)^49^, which examines self-perceived cognitive difficulties in attention and concentration, retrospective and prospective memory, planning and organization, and executive functioning on a 5-point Likert scale (0 = never, 4 = almost always, with higher scores reflecting greater perceived cognitive impairment). These measures served to phenotype the neuropsychological response and allow for correlation with molecular miRNA trajectories.

### Plasma collection and processing

Peripheral blood samples were collected by venipuncture into 10 ml K_2_EDTA BD Vacutainer tubes (Becton Dickinson, Franklin Lakes, NJ, USA). To minimize pre-analytical variability, samples were processed within 1 h of collection. A two-step centrifugation protocol was implemented to obtain cell-free plasma: first, blood was centrifuged at 450 × g for 10 min at room temperature (22–25 °C) to separate plasma from cellular components. The supernatant was then transferred to a fresh tube and centrifuged at 2500 × g for 15 min (Eppendorf 5702, Hamburg, Germany) to remove residual platelets and cellular debris. Plasma aliquots (500 µl) were immediately stored at − 80 °C until RNA isolation.

### RNA isolation and quality control

Total RNA, enriched for small RNAs, was isolated from 200 µl of thawed plasma using the miRNeasy Serum/Plasma Kit (Qiagen, Hilden, Germany) according to the manufacturer’s protocol. Prior to isolation, a stringent quality control step was performed to exclude hemolysis, a critical confounder in plasma miRNA analysis. Free hemoglobin absorbance was measured at 414 nm (NanoDrop™), and only samples meeting the inclusion criteria (A_414nm_ < 0.2) were processed (Suppl. Figure 1 A). During the lysis step, 1 µl of the synthetic C. elegans miRNA mimic cel-miR-39-3p (Qiagen) was added to each sample as an exogenous spike-in control to monitor the efficiency of RNA extraction and downstream enzymatic reactions. Total RNA was eluted in 15 µl of RNase-free water.

### cDNA synthesis and quantitative PCR (qPCR)

Expression levels of a targeted panel of 38 miRNAs were quantified using a two-step RT-qPCR protocol. Due to the low RNA concentration in plasma, a fixed-volume input strategy was employed. First, polyadenylation was performed using 7.5 µl of total RNA eluate, 1.5 U of *E. coli* poly(A) polymerase I (AM2030, Thermo Fisher Scientific), and 1 mM ATP (R0441, Thermo Fisher Scientific) at 37 °C for 60 min, followed by enzyme heat inactivation at 65 °C for 10 min. Second, 12 µl of the polyadenylated RNA was used as a template for reverse transcription in a 50 µl reaction with the High-Capacity cDNA Reverse Transcription Kit (Ref. 4368814, Thermo Fisher Scientific). The reaction mix was prepared according to manufacturer’s recommendations, containing 100 nM of a custom degenerate oligo-dT adapter primer (5′-GCACGCTACGACCTCAGAGTCACTATGGCACTTTTTTTTTTTTTTTTVN-3′, V = G/A/C and N = G/A/T/C), 1X RT Buffer, 1 mM dNTPs, and 2.5 U/µl MultiScribe™ Reverse Transcriptase. The reaction was incubated at 25 °C for 10 min, followed by 37 °C for 120 min, and enzyme inactivation at 85 °C for 5 min. qPCR was performed on an Applied Biosystems 7500 Real-Time PCR platform (Thermo Fisher) in compliance with MIQE guidelines^43,44^. Reactions were run in triplicate using PowerTrack™ SYBR Green Master Mix (Thermo Fisher Scientific) and miRNA-specific forward primers containing 2 µl of cDNA template, 1X PowerTrack™ SYBR Green Master Mix (Thermo Fisher Scientific), and 400 nM of each primer in 10 µl. The cycling conditions included an initial activation at 95 °C for 2 min, followed by 40 cycles of 95 °C for 15 s and 60 °C for 60 s, ending with a melt curve analysis to confirm amplicon specificity.

### Data normalization and analysis

Data pre-processing involved excluding replicates with a standard deviation (SD) > 0.3 Cq. Technical consistency across samples was verified using the exogenous spike-in cel-miR-39-3p (Suppl. Figure 1B). Relative expression was quantified using the 2^ΔΔCq^ method, which represents the relative fold-change in miRNA levels. To ensure robust normalization, the stability of candidate reference genes was evaluated using the NormFinder algorithm, which identified hsa-miR-151b and hsa-miR-27b-5p as the optimal reference pair (stability value = 0.074). The stability of this pair was further validated by a 3-way ANOVA showing no significant effects of experimental conditions (*p* > 0.05) (Suppl. Figure 1 C). For the relative quantification, each sample’s Cq value was first normalized to the mean of this reference pair to obtain the ΔCq. Subsequently, the ΔΔCq was calculated by normalizing each post-treatment sample (T_1_) and each individual pre-treatment sample (T_0_) to the mean of the entire pre-treatment group (used as the control calibrator). All primer sequences are listed in Suppl. Table 1.

### Bioinformatic target prediction and functional enrichment

To delineate the biological mechanisms involved in modulated miRNAs, a systems biology approach was applied. Putative gene targets were identified using a multi-algorithmic strategy integrating seven databases (miRWalk, MicroT, mirabel, miRDB, miRTargetLink, TargetScan, and miRTarBase). We retained both experimentally validated targets and predicted targets (binding probability > 0.90) in the 3’ untranslated region (UTR), 5’ UTR, and coding DNA sequence (CDS) regions. To minimize false positives, a stringent consensus filter was applied: only targets experimentally validated (miRTarBase) or predicted by ≥ 5 algorithms were retained.

Functional annotation of the consensus target lists was performed through Over-Representation Analysis (ORA) using the web-based GEne SeT AnaLysis Toolkit (WebGestalt; http://www.webgestalt.org). We used the protein-coding genome background as the reference set. Enrichment for Kyoto Encyclopedia of Genes and Genomes (KEGG) pathways^53–55^ and Gene Ontology (GO) Biological Processes was determined using a hypergeometric test with Benjamini-Hochberg FDR correction (FDR < 0.05). Only KEGG pathways and GO terms containing at least five of our target genes were retained (Suppl. Excel File 1). The subsequent analysis focuses on the most statistically significant pathways and those with established biological relevance to MDD pathophysiology and neuroplasticity, based on cross-referencing with published literature. Redundant or hierarchical parent/child GO terms were consolidated to avoid over-representation. Network interaction maps were generated using Cytoscape to visualize the functional convergence of the identified pathways, where nodes represented functional terms scaled by gene set size, and edge thickness was proportional to the degree of gene overlap.

### Statistical analyses

Data are presented as the mean ± standard error of the mean (SEM). To ensure transparency of data dispersion and mitigate the influence of the exploratory sample size, individual data points are displayed in all graphical representations. Normality of data distribution was assessed using the Shapiro-Wilk test, and outliers were identified and excluded using the Robust Regression and Outlier Removal (ROUT) method with a strict Q-coefficient of 1%. Descriptive statistics were generated for all sociodemographic and clinical characteristics. To mitigate the limitations of the exploratory sample size, the statistical strategy prioritized longitudinal within-subject comparisons, leveraging the paired nature of the design to control for inter-individual variability and maximize statistical power. All analyses were performed using SPSS (version 30, IBM Corp.) and GraphPad Prism (version 10.4.1).

For clinical and functional outcomes involving three time-points (T_0_, T_1_, T_2_), the non-parametric Friedman test was used to robustly assess temporal trajectories within each intervention group, accounting for the non-normal distribution of psychometric variables. Significant main effects were followed by Dunn’s post-hoc tests for pairwise comparisons. Between-group comparisons at baseline were conducted using independent samples t-tests for normally distributed variables and Mann–Whitney U tests for non-normally distributed data.

For the molecular analysis of circulating miRNAs, the primary analysis of miRNA levels relied on a two-way ANOVA with repeated measures. The model included time (pre- vs. post-treatment) as the within-subject factor and group (INCREM vs. PSYCHOED) as the between-subject factor. This model allowed us to test the interaction between group and time followed by Šídák’s multiple comparisons test to correct for family-wise error rate (FWER). For simple paired comparisons (e.g., within-group changes from T_0_ to T_1_), paired Student’s t-tests or Wilcoxon signed-rank tests were applied based on normality.

All tests were two-tailed, with statistical significance set at *p* < 0.05. Where applicable, effect sizes were estimated to determine the magnitude of the observed changes beyond statistical significance. These were partial eta squared (ƞ_p_^2^) for ANOVA models; Cohen’s *d* (d_av_) for parametric t-tests; the rank-biserial correlation (r_rb_) for non-parametric comparisons (Mann-Whitney U and Wilcoxon signed-rank tests); and Kendall´s W for Friedman test. A comprehensive summary of all test statistics, including test values, degrees of freedom, and effect size estimates, is provided in Suppl. Excel File 2.

## Results

### Sociodemographic and clinical characteristics

A total of 22 patients diagnosed with MDD in partial remission were included in the study. Of these, 15 were randomized to the INCREM intervention and 7 to the PSYCHOED condition. As detailed in Table 1A, no statistically significant differences were found between the two groups regarding baseline sociodemographic or clinical variables, ensuring the comparability of the study arms prior to intervention.

### Identification of therapy-specific circulating miRNA profile

**Fig. 1:**
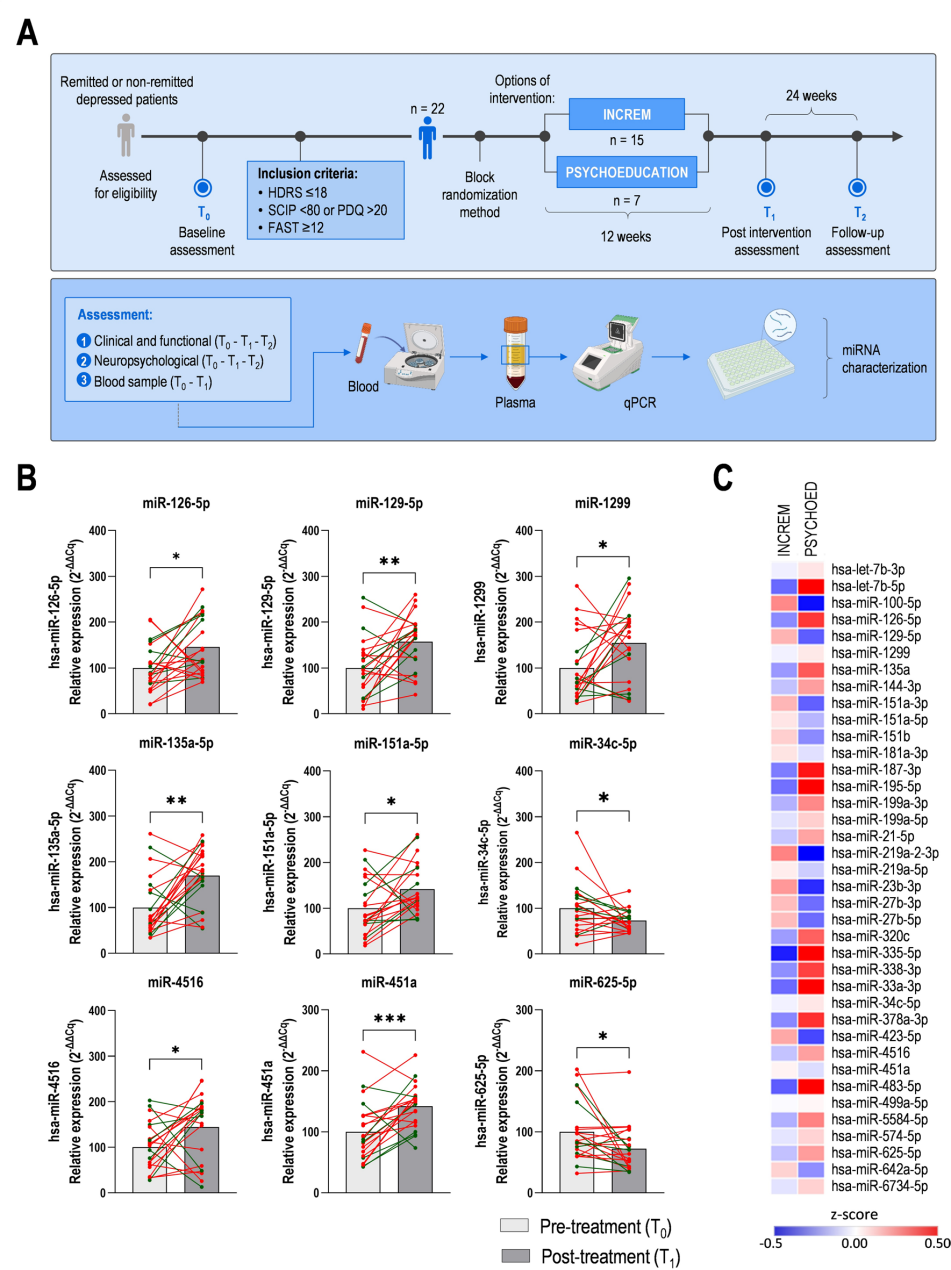
Study design and identification of plasma miRNAs modulated by psychotherapy in MDD patients. (**A**) Schematic of the experimental design. A panel of 36 miRNAs, which were previously found to be dysregulated in the post-mortem dorsolateral prefrontal cortex (dlPFC) of MDD patients, was analyzed in plasma samples from a separate cohort of MDD patients before (pre-treatment, T_0_) and after (post-treatment, T_1_) intervention with either integral cognitive remediation (INCREM, *n* = 15) or psychoeducation (PSYCHOED, *n* = 7). (**B**) Bar graphs showing 9 miRNA levels that were significantly modulated by psychotherapy (combined INCREM and PSYCHOED groups, indicated in red and green, respectively) compared to baseline (pre-treatment). (**C**) Hierarchical clustering heat-map displaying the mean z-score expression of the 36 miRNAs for each group (INCREM- and PSYCHOED). Color intensity represents the relative expression levels, where red indicates higher expression (positive z-scores) and blue indicates reduced expression (negative z-scores). Data are presented as mean ± SEM. *p < 0.05, **p < 0.01, ***p < 0.001 when comparing the values before and after intervention (detailed statistical analysis in Supplementary Excel File 2).

To determine the molecular footprint of each intervention, we quantified the levels of 38 candidate miRNAs in paired pre- and post-treatment plasma samples (Fig. 1A). This panel of miRNAs was selected based on our previous transcriptomic sequencing and qPCR validation in postmortem dlPFC samples of patients with MDD (male/female)^43^, aiming to translate central dysregulation findings to peripheral biomarkers (Table 2 and Suppl. Figure 2). Initial analysis of the pooled cohort (combining INCREM and PSYCHOED), showed that the miRNA panel responded to psychological intervention. Significant temporal modulation was observed in 9 miRNAs, including the upregulation of miR-126-5p (*p* < 0.05; r_rb_ = 0.52), miR-129-5p (*p* < 0.01; Cohen’s *d* = 0.90), miR-1299 (*p* < 0.05; r_rb_ = 0.38), miR-135a-5p (*p* < 0.01; r_rb_ = 0.62), miR-151a-5p (*p* < 0.05; r_rb_ = 0.47), miR-4516 (*p* < 0.05; r_rb_ = 0.38), and miR-451a (*p* < 0.001; Cohen’s *d* = 0.70). Conversely, the levels of two miRNAs, miR-34c-5p (*p* < 0.05; Cohen’s *d* = -0.69) and miR-625-5p (*p* < 0.05; Cohen’s *d* = 0.44), decreased significantly (Fig. 1B).

**Table 1:** Participant characteristics and functional-cognitive outcomes.

A. Baseline sociodemographic and clinical characteristics: whole sample and intervention groups					
	WHOLE SAMPLE (*n* = 22)	INCREM (*n* = 15)	PSYCHOED (*n* = 7)	F	*p*-value
Age, *years*	50.36 (± 1.94)	50.8 (± 1.84)	49.43 (± 4.90)	3.4	0.750
Years of schooling, *n*^*#*^	16.14 (± 0.62)	15.87 (± 0.74)	16.71 (± 1.19)	43.0	0.535
Estimated IQ, *T-score*	61.50 (± 1.84)	63.07 (± 2.15)	58.14 (± 3.38)	0.12	0.221
Age at illness onset, *years*	34.95 (± 2.75)	35.53 (± 3.53)	33.71 (± 4.50)	0.13	0.766
Depressive episodes, *n*^*#*^	4.91 (± 1.60)	5.40 (± 2.31)	3.86 (± 1.12)	39.5	0.368
Clinical stability, *months*^*#*^	100.64 (± 34.89)	112.33 (± 44.67)	75.57 (± 57.10)	40.0	0.407
Duration of illness, *years*	15.40 (± 10.52)	15.27 (± 10.52)	15.71 (± 11.37)	52.0	0.972
ATHF, *total score*^*#*^	2.64 (± 0.21)	2.6 (± 0.3)	2.71 (± 0.19)	50.5	0.891
HDRS-17, *total score*	11.05 (± 1.09)	10.8 (± 1.31)	11.57 (± 2.10)	0.04	0.750

B. Functional and cognitive outcomes at baseline, post-intervention and at follow-up assessment					
	Baseline	Post	Follow	F	*p-value*
INCREM					
FAST, *total score*	28.86 (± 2.40)	21.21 (± 2.74)	14.0 (± 3.19)	12.69	0.002**
SCIP, *total score*	73.00 (± 3.44)	82.79 (± 2.41)	82.86 (± 3.20)	13.0	0.002**
PDQ, *total score*	41.93 (± 2.92)	33.93 (± 2.97)	32.43 (± 3.66)	8.71	0.013*
PSYCHOED					
FAST, *total score*	33.67 (± 5.70)	26.17 (± 6.62)	17.67 (± 6.21)	10.33	0.006**
SCIP, *total score*	68.33 (± 3.46)	76.83 (± 3.78)	74.83 (± 5.46)	4.26	0.119
PDQ, *total score*	48.33 (± 3.67)	42.33 (± 5.18)	39.83 (± 7.22)	5.33	0.069

IQ: Intelligence Quotient, ATHF: Antidepressant Treatment History Questionnaire, HDRS-17: 17-item Hamilton Depression Rating Scale, FAST: Functioning Assessment Short Test, SCIP: Screening for Cognitive Impairment in Psychiatry, PDQ: Perceived Deficit Questionnaire. ^*#*^Comparison by Mann-Whitney U test. Data are presented as mean ± SEM. *p < 0.05, **p < 0.01 for within-group changes over time.

**Table 2:** Panel of analyzed miRNAs used in the study.

miRNA	miRbase (ACC. No)	Locus	Fold change	*p*-value
hsa-let-7b-3p	MIMAT0004482	chr22: 46,113,686–46,113,768	0.7921	0.0982
hsa-let-7b-5p	MIMAT0000063	chr22: 46,113,686–46,113,768	1.036	0.7943
hsa-miR-100-5p	MIMAT0000098	chr11: 122,152,229–122,152,308	0.7886	0.0869
hsa-miR-126-5p	MIMAT0000444	chr9: 136,670,602–136,670,686	1.453	0.0156*
hsa-miR-129-5p	MIMAT0000242	chr7: 128,207,872–128,207,943	1.571	0.0040**
hsa-miR-1299	MIMAT0005887	chr9: 40,929,010–40,929,092	1.540	0.0359*
hsa-miR-135a-5p	MIMAT0000428	chr9: 40,929,010–40,929,092	1.701	0.0017**
hsa-miR-144-3p	MIMAT0000436	chr17: 28,861,533–28,861,618	1.064	0.4589
hsa-miR-151a-3p	MIMAT0000757	chr8: 140,732,564–140,732,653	1.177	0.2581
hsa-miR-151a-5p	MIMAT0004697	chr8: 140,732,564–140,732,653	1.418	0.0141*
hsa-miR-151b	MIMAT0010214	chr14: 100,109,419–100,109,514	N/A	N/A
hsa-miR-181a-3p	MIMAT0000270	chr1: 198,859,044–198,859,153	1.095	0.7593
hsa-miR-187-3p	MIMAT0000262	chr18: 35,904,818–35,904,926	0.8559	0.2187
hsa-miR-195-5p	MIMAT0000461	chr17: 7,017,615–7,017,701	1.297	0.0587
hsa-miR-199a-3p	MIMAT0000232	chr19: 10,817,426–10,817,496	0.8081	0.3169
hsa-miR-199a-5p	MIMAT0000231	chr19: 10,817,426–10,817,496	1.206	0.1375
hsa-miR-21-5p	MIMAT0000076	chr17: 59,841,266–59,841,337	0.8537	0.0760
hsa-miR-219a-2-3p	MIMAT0004675	chr9: 128,392,618–128,392,714	0.9437	0.7404
hsa-miR-219a-5p	MIMAT0000276	chr6: 33,207,835–33,207,944	0.9307	0.6556
hsa-miR-23b-3p	MIMAT0000276	chr9: 95,085,208–95,085,304	1.119	0.6327
hsa-miR-27b-3p	MIMAT0000419	chr9: 95,085,445–95,085,541	1.371	0.0566
hsa-miR-27b-5p	MIMAT0004588	chr9: 95,085,445–95,085,541	N/A	N/A
hsa-miR-320c	MIMAT0005793	chr18: 21,683,518–21,683,589	0.9815	0.1765
hsa-miR-335-5p	MIMAT0000765	chr7: 130,496,111–130,496,204	1.241	0.2290
hsa-miR-338-5p	MIMAT0004701	chr17: 81,125,883–81,125,949	0.8831	0.4628
hsa-miR-33a-3p	MIMAT0004506	chr22: 41,900,944–41,901,012	1.200	0.1375
hsa-miR-34c-5p	MIMAT0000686	chr11: 111,513,439–111,513,515	0.7313	0.0435*
hsa-miR-378a-3p	MIMAT0000732	chr5: 149,732,825–149,732,890	0.7931	0.1762
hsa-miR-423a-5p	MIMAT0004748	chr17: 30,117,079–30,117,172	1.186	0.1649
hsa-miR-4516	MIMAT0019053	chr16: 2,133,119–2,133,204	1.444	0.0391*
hsa-miR-451a	MIMAT0001631	chr17: 28,861,369–28,861,440	1.420	0.0005***
hsa-miR-483-5p	MIMAT0004761	chr11: 2,134,134–2,134,209	1.208	0.3369
hsa-miR-499a-5p	MIMAT0002870	chr20: 34,990,376–34,990,497	0.8296	0.3209
hsa-miR-5584-5p	MIMAT0022283	chr1: 44,545,493–44,545,552	0.7567	0.2615
hsa-miR-574-5p	MIMAT0004795	chr4: 38,868,032–38,868,127	0.7863	0.2285
hsa-miR-625-5p	MIMAT0003294	chr14: 65,471,102–65,471,186	0.7227	0.0187*
hsa-miR-642a-5p	MIMAT0003312	chr19: 45,674,928–45,675,024	1.270	0.1317
hsa-miR-6734-5p	MIMAT0027369	chr1: 43,364,648–43,364,715	0.9644	0.9493

Significantly changed miRNAs in the plasma of patients with MDD after psychotherapy intervention. For normalization, expression stability of the 38-miRNA panel was assessed using the NormFinder algorithm, which identified hsa-miR-151b and hsa-miR-27b-5p as the most stable reference pair. N/A Not applicable. *p < 0.05, **p < 0.01, ***p < 0.001 pre-treatment vs. post-treatment.

Hierarchical clustering analysis of the modulated miRNAs revealed distinct expression patterns that differentiated the INCREM group from the PSYCHOED group (Fig. 1C and Suppl. Figure 3). Subsequent differential expression analysis confirmed intervention-specific profiles. Significant increases were notably observed in the levels of miR-129-5p (*p* < 0.05; Cohen’s *d* = 1.02), miR-135a-5p (*p* < 0.01; r_rb_ = 0.45), miR-151a-5p (*p* < 0.05; r_rb_ = 0.62), miR-4516 (*p* < 0.05; Cohen’s *d* = 0.95), and miR-451a (*p* < 0.001; Cohen’s *d* = 1.75), as well as decreases in let-7b-3p (*p* < 0.05; r_rb_ = 0.62) and miR-100-5p (*p* < 0.05; Cohen’s *d* = -0.99) in the plasma of MDD patients when comparing pre- and post-INCREM treatment (Suppl. Figure 4). In contrast, a distinct profile involving the upregulation of only miR-126-5p (*p* < 0.05; Cohen’s *d* = 1.65) and miR-195-5p (*p* < 0.05; Cohen’s *d* = 1.51) was observed following PSYCHOED treatment in MDD patients (Suppl. Figure 5). To further validate these findings, we integrated the treatment-responsive miRNAs into a composite biomarker index for each intervention. This multiplexed approach confirmed highly significant longitudinal trajectories for both groups, effectively reducing individual noise and reinforcing the robustness of the identified miRNA signatures (Suppl. Figure 6).

### Functional pathway analysis of intervention-specific miRNA profiles

**Fig. 2:**
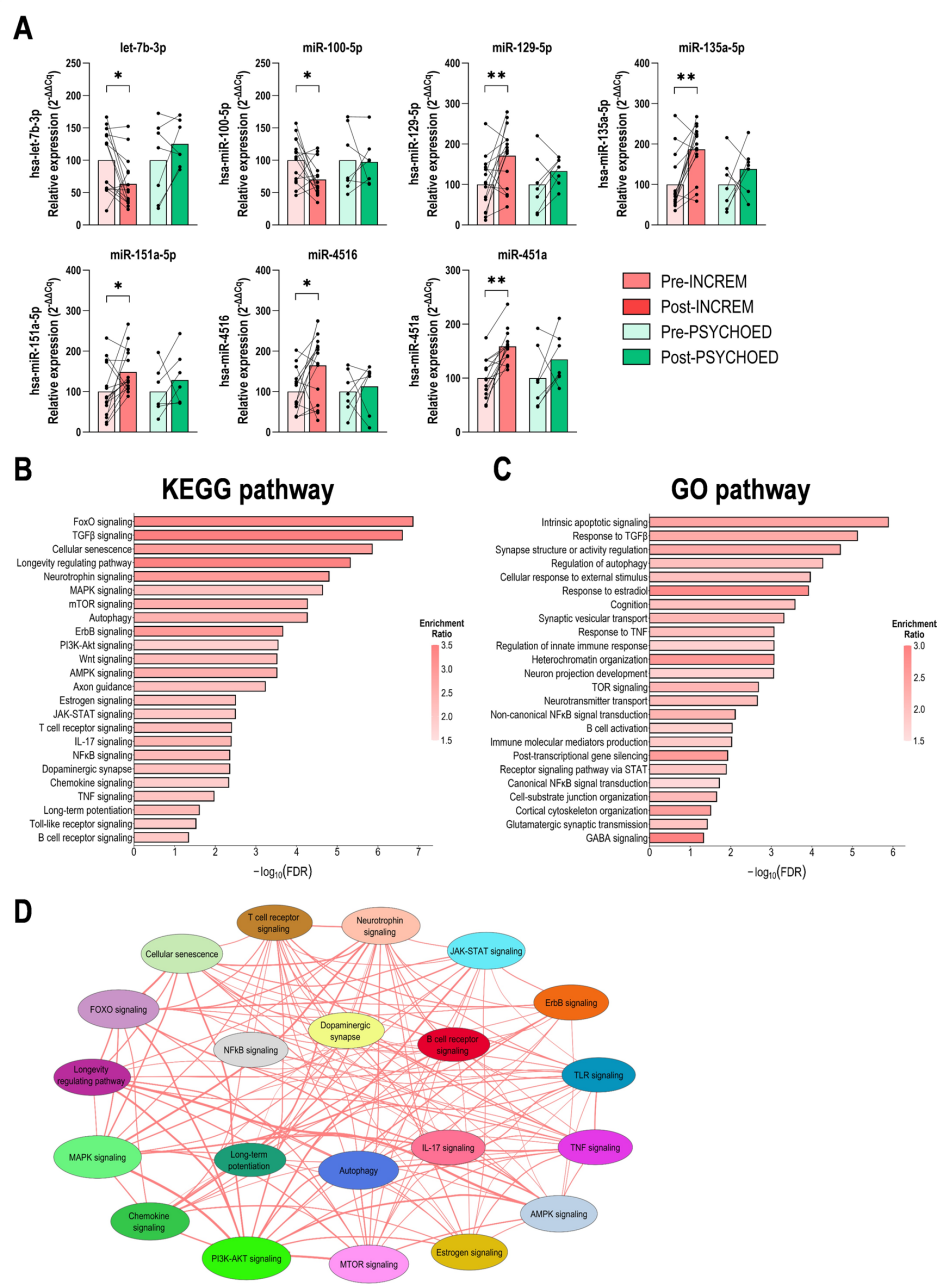
The functional targetome of circulating miRNAs specifically regulated by INCREM. (**A**) Relative plasma levels of the 7 miRNAs significantly modulated by integral cognitive remediation (INCREM, *n* = 15) intervention. The graphs show the significant changes in let-7b-3p, miR-100-5p, miR-129-5p, miR-135a-5p, miR-151a-5p, miR-4516, and miR-451a comparing pre-INCREM vs. post-INCREM. (**B**) Kyoto Encyclopedia of Genes and Genomes (KEGG)-based pathway analysis of the predicted target genes of the dysregulated miRNAs. The bar chart shows the enrichment ratio of the most significantly enriched pathways. (**C**) Gene Ontology (GO) analysis for the biological process category. The bar chart displays the enrichment ratio of the most significant processes associated with the predicted miRNA target genes. (**D**) Enrichment network visualizing the relationships among the identified KEGG functional pathways. Each node represents an enriched term, color-coded by functional cluster. The connecting edges indicate the degree of overlap based on shared genes between the terms. Data are presented as mean ± SEM. *p < 0.05, **p < 0.01 when comparing the values before and after intervention (detailed statistical analysis in Supplementary Excel File 2).

Next, a two-way ANOVA analysis was conducted to confirm the differential effects of both psychotherapies on plasma miRNA levels. Šídák’s multiple comparisons test revealed a significant time effect for seven miRNAs: let-7b-3p (*p* < 0.05; Cohen’s *d* = -0.85), miR-100-5p (*p* < 0.05; Cohen’s *d* = -0.99), miR-129-5p (*p* < 0.01; Cohen’s *d* = 1.02), miR-135a-5p (*p* < 0.01; Cohen’s *d* = 1.31), miR-151a-5p (*p* < 0.05; Cohen’s *d* = 0.82), miR-4516 (*p* < 0.05; Cohen’s *d* = 0.95), and miR-451a (*p* < 0.01; Cohen’s *d* = 1.75) in patients receiving cognitive remediation (INCREM group). Importantly, these miRNAs were not significantly altered in the PSYCHOED group (Fig. 2A).

To explore the potential biological implications of the INCREM-specific miRNA profile, a functional enrichment analysis was performed on the consensus-predicted target genes (Fig. 2B-D). KEGG pathway analysis revealed a significant over-representation of target genes in pathways regulating neuroplasticity, cellular signaling, and synaptic function (Fig. 2B,D). The most significant of these were neurotrophin signaling (FDR = 1.59 × 10⁻⁵), axon guidance (FDR = 5.85 × 10⁻^4^), long-term potentiation (FDR = 0.024), and the dopaminergic synapse (FDR = 4.40 × 10^− 3^). Furthermore, a significant enrichment was observed in critical intracellular signaling cascades that are known to regulate neuronal survival and plasticity, such as TGFβ signaling (FDR = 2.50 × 10⁻⁷), FOXO signaling (FDR = 1.37 × 10⁻⁷), PI3K-Akt signaling (FDR = 2.86 × 10⁻⁴), mTOR signaling (FDR = 5.42 × 10⁻⁵), MAPK signaling (FDR = 2.29 × 10⁻⁵), and NFκB signaling (FDR = 4.40 × 10^− 3^). GO analysis of biological processes further supported these findings, suggesting an over-representation of terms associated with synaptic function. The most relevant enriched terms included the regulation of synapse structure and activity (FDR = 4.88 × 10⁻^4^), neuron projection development (FDR = 8.76 × 10⁻⁴), and cognition (FDR = 2.59 × 10⁻⁴). Consistent with a hypothesized role in synaptic communication, the analysis revealed a significant enrichment in both excitatory and inhibitory signaling processes. Specifically, two pathways were identified: the glutamatergic synaptic transmission pathway (GO, FDR = 0.037) and the long-term potentiation pathway (KEGG, FDR = 0.024); the latter is a primary cellular mechanism for learning and memory. However, this was offset by the enrichment of the GABA signaling pathway (FDR = 0.046), suggesting that INCREM-modulated miRNAs could contribute to restoring the balance between excitation and inhibition (Fig. 2C), a key mechanism compromised in MDD pathophysiology.

**Fig. 3:**
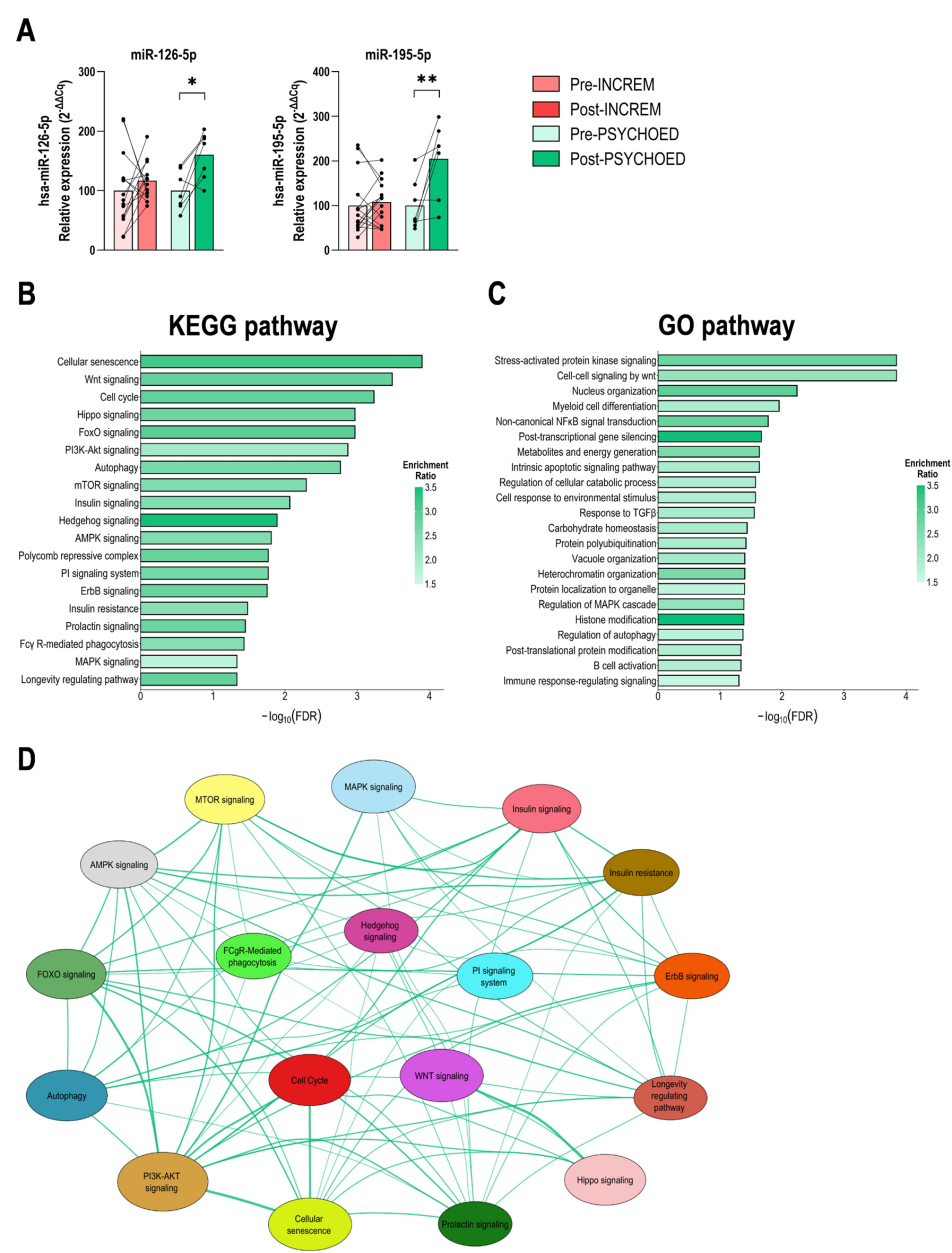
The functional targetome of circulating miRNAs specifically regulated by PSYCHOED. (**A**) Relative plasma levels of the 2 miRNAs significantly modulated by psychoeducation (PSYCHOED, *n* = 7) intervention. The graphs show the significant changes in miR-126-5p and miR-195-5p comparing pre-PSYCHOED vs. post-PSYCHOED. (**B**) Kyoto Encyclopedia of Genes and Genomes (KEGG)-based pathway analysis of the predicted target genes of the dysregulated miRNAs. The bar chart shows the enrichment ratio of the most significantly enriched pathways. (**C**) Gene Ontology (GO) analysis for the biological process category. The bar chart displays the enrichment ratio of the most significant processes associated with the predicted miRNA target genes. (**D**) Enrichment network visualizing the relationships among the identified KEGG functional pathways. Each node represents an enriched term, color-coded by functional cluster. The connecting edges indicate the degree of overlap based on shared genes between the terms. Data are presented as mean ± SEM. *p < 0.05, **p < 0.01 when comparing the values before and after intervention (detailed statistical analysis in Supplementary Excel File 2).

In contrast, a two-way ANOVA followed by Šidák’s multiple comparisons test revealed significant time effects for both miR-126-5p (*p* < 0.05; Cohen’s *d* = 1.65) and miR-195-5p (*p* < 0.01; Cohen’s *d* = 1.51) within the PSYCHOED group, though not within the INCREM group **(**Fig. 3A). Functional analysis showed that their predicted targets converge on general cellular homeostasis and cell fate decisions (Fig. 3B-D). The most significantly enriched KEGG pathways were cellular senescence (FDR = 1.26 × 10^− 4^), Wnt signaling (FDR = 3.26 × 10^− 4^), the cell cycle (FDR = 5.83 × 10^− 4^), the Hippo signaling (FDR = 1.07 × 10^− 3^), and the PI3K-Akt signaling (FDR = 1.34 × 10^− 3^) (Fig. 3B and D). GO analysis also indicated enrichment in pathways related to cellular communication and stress responses, such as stress-activated protein kinase signaling (FDR = 1.42 × 10^− 4^), cell-cell signaling by Wnt (FDR = 1.42 × 10^− 4^), nucleus organization (FDR = 5.71 × 10^− 3^), and the intrinsic apoptotic signaling pathway (FDR = 0.023) (Fig. 3C). Unlike the INCREM profile, no significant enrichment was observed in pathways directly related to cognition or synaptic transmission. These results suggest potential differences in the molecular signatures underlying the functional effects of these two types of non-pharmacological intervention.

### Functional and cognitive trajectories following psychological interventions

**Fig. 4:**
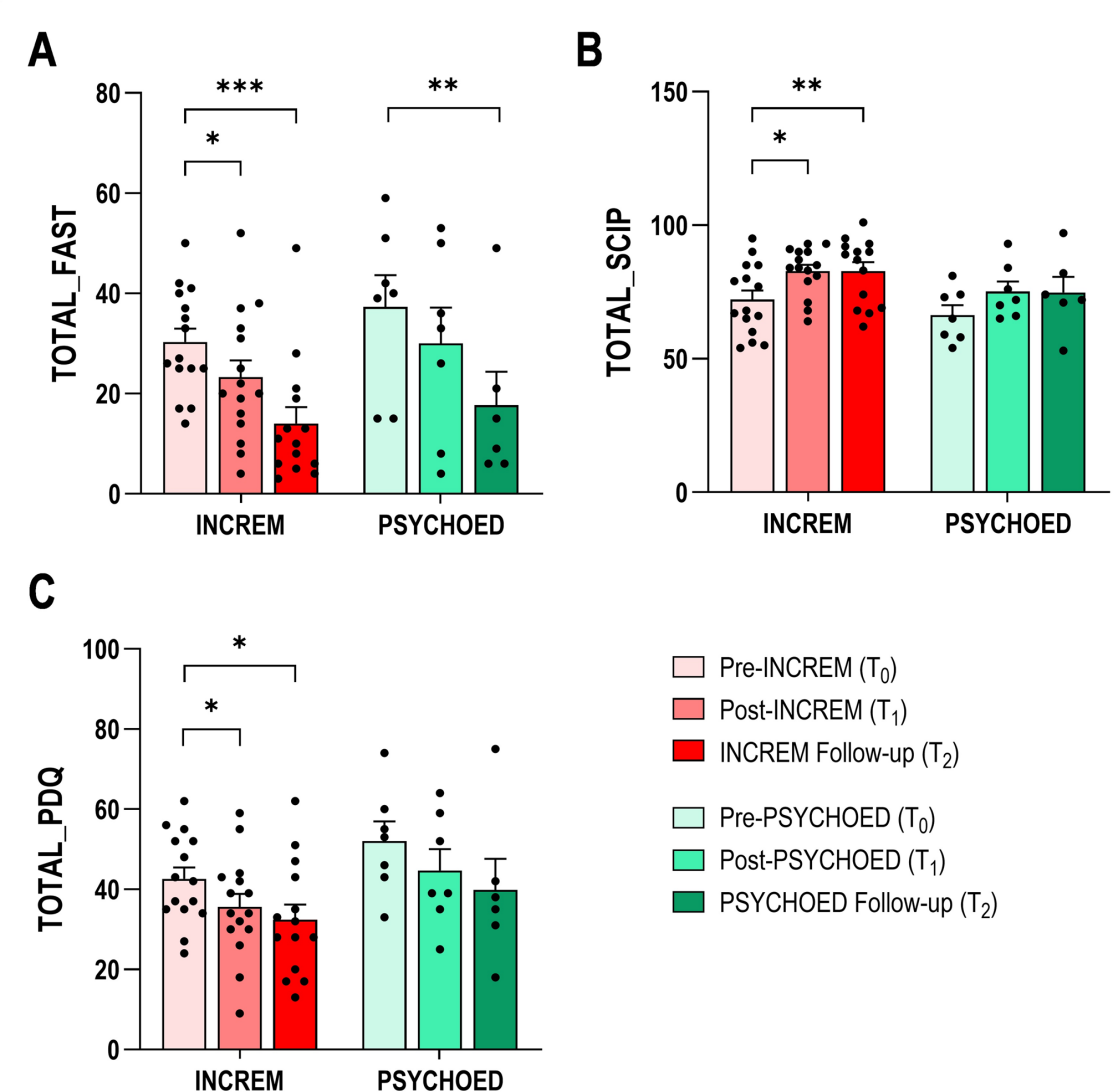
INCREM therapy, but not PSYCHOED, improves cognitive function in MDD patients. Bar graphs showing cognitive performance in MDD patients before (pre-treatment, T_0_), after (post-treatment, T_1_), and follow-up (T_2_) intervention with either integral cognitive remediation (INCREM, *n* = 15) or psychoeducation (PSYCHOED, *n* = 7). **(A)** Psychosocial dysfunction (FAST) **(B)** Objective cognitive performance (SCIP) **(C)** Perceived cognitive deficits (PDQ). Data are presented as mean ± SEM. *p < 0.05, **p < 0.01, and ***p < 0.001when comparing the values before (T_0_) and after intervention (T_1_ and T_2_). Detailed statistical analysis in Supplementary Excel File 2.

Table 1B summarizes the longitudinal functional and cognitive outcomes across assessment points (T_0_ pre-treatment, T_1_ post-treatment, and T_2_ follow-up). Analysis of psychosocial functioning (FAST) revealed that while both interventions were beneficial, they exhibited distinct temporal kinetics (Fig. 4A). The INCREM group showed a rapid and sustained response, with significant reductions in functional impairment observed immediately post-treatment (T_1_, *p* < 0.05, r_rb_ = 0.48) and maintained at follow-up (T_2_, *p* < 0.001, r_rb_ = 0.70). In contrast, the PSYCHOED group displayed a delayed therapeutic effect, reaching statistical significance only at the 6-month follow-up (T_2_, *p* < 0.01, r_rb_ = 0.92) compared to T_0_ pre-treatment.

Regarding cognitive outcomes, the interventions elicited different responses. The INCREM group showed significant and sustained improvements in objective cognitive performance (SCIP) at both T_1_ (*p* < 0.05, r_rb_ = 0.48) and T_2_ (*p* < 0.001, r_rb_ = 0.64), alongside significant reductions in perceived cognitive deficits (PDQ) at T_1_ (*p* < 0.05, r_rb_ = 0.50) and T_2_ (*p* < 0.05, r_rb_ = 0.46). In contrast, the PSYCHOED intervention produced no statistically significant changes in either SCIP or PDQ scores at any assessment point (Fig. 4B and C). These cognitive data are consistent with bioinformatic findings, suggesting that INCREM-induced modulation of specific miRNAs related to neuroplasticity and synaptic function pathways may translate into quantifiable improvements in higher-order cognitive functions. Conversely, the delayed functional improvement observed in the PSYCHOED group is related with a mechanism based on systemic homeostasis and stress resilience, rather than direct neural remodeling.

## Discussion

In this exploratory study, we provide preliminary molecular evidence that psychological interventions, specifically INCREM and PSYCHOED, elicit distinct biological responses in patients with MDD. Despite the small sample size, the identification of non-overlapping circulating miRNA profiles for each intervention suggests that the therapeutic effects are specific to the content and target of the intervention, rather than general. This finding represents a conceptual and hypothesis-generating step forward, supporting the transition from viewing psychological interventions through a purely behavioral framework to recognizing their measurable biological basis. While previous neuroimaging research has demonstrated that psychotherapy (e.g., CBT) induces neuroplasticity and functional connectivity changes in key brain networks^19,24,56^, our pilot results extend this understanding to the peripheral molecular level. By directly comparing two active interventions, this exploratory analysis suggests that INCREM and PSYCHOED engage distinct biological machinery. These initial findings provide a biological rationale for the future development of precision psychiatry strategies, where therapy selection could be guided not only by symptom patterns but also by the patient’s underlying molecular profile.

Identifying a seven-miRNA profile specific to the INCREM response (comprising let-7b-3p, miR-100-5p, miR-129-5p, miR-135a-5p, miR-151a-5p, miR-4516, and miR-451a) suggests that cognitive improvement might be linked to defined molecular mechanisms rather than non-specific effects. The INCREM-induced upregulation of miR-129-5p and miR-135a-5p is noteworthy as it points to a potential molecular convergence with established antidepressant pharmacological mechanisms. The expression of miR-129-5p is reduced in post-mortem dlPFC samples and in brain extracellular vesicles obtained from individuals with MDD^43,57^, but its plasma levels increase following 12-week of SSRI escitalopram treatment^58^. In mouse models, downregulation of miR-129-5p accompanies depressive-like phenotypes, while its overexpression in hippocampus mitigates such behaviors by reducing microglial activation and neuroinflammation dependent of NFκB pathway^59^. Similarly, miR-135a is a well-established regulator of stress resilience and the antidepressant response, acting on the serotonergic system by targeting the serotonin transporter (SERT) and the 5-HT_1A_ autoreceptor^31,60^. Its levels have been shown to decrease in plasma and post-mortem brain samples from MDD patients, as well as in mouse models exhibiting depressive-like behavior^31,61,62^. In contrast, 8-week pharmacological treatment with duloxetine or 12-week treatment with venlafaxine increases its plasma levels^63,64^. Beyond its role in the serotonergic system, miR-135a contributes to synaptic plasticity and memory formation. Interestingly, a 3-month CBT programme was also found to increase blood miR-135a levels in MDD patients^31^. While CBT focuses on cognitive restructuring and INCREM targets functional neurocognitive deficits, the fact that there is a parallel increase in miR-135a-5p following both interventions suggests that they have a shared biological basis. Taken together, these findings lead us to hypothesize that INCREM activates molecular pathways that are similar to those involved in pharmacological and psychological antidepressant strategies, likely to be mediated by enhanced serotonergic signaling and synaptic plasticity.

Furthermore, the significant increase in miR-451a following INCREM suggests a potential molecular link to synaptic dysfunction in MDD. This finding aligns with substantial evidence showing that the miR-451a level is reduced in the serum and cerebrospinal fluid (CSF) of patients with MDD, often correlating with the depressive symptom severity^65,66^. Although there is some variability across cohorts, miR-451a is a promising therapeutic marker as baseline levels predict antidepressant efficacy, and plasma expression is restored following 8-week paroxetine treatment^65,67^. Preclinical models provide mechanistic support of miR-451a for the goals of cognitive remediation. In mouse models of chronic stress-induced depression, miR-451a is significantly downregulated in both serum and the medial prefrontal cortex (mPFC), concurrently with dendritic spine loss. Overexpression of miR-451a in the mPFC of these mice reverses this structural damage and improves depressive-like phenotype by inhibiting corticotrophin-releasing factor receptor 1 (CRF-1) expression^68^. This establishes a mechanistic bridge between the change in a circulating biomarker and the restoration of structural neuroplasticity in the brain, a goal of pro-cognitive treatments. Convergent evidence from other neurological conditions further underscores the importance of miR-451a in the relationship between mood and cognition. In patients with Alzheimer’s disease (AD), reduced CSF miR-451a levels were associated with both lower cognitive scores and higher depression ratings^69^. The same study also found reduced miR-451a levels in neuronal and microglial cultures from AD-like APP/PS1 transgenic mice, while its overexpression in the mPFC improved behavioral and pathological alterations, including long-term memory deficits, depression-like phenotype, and NFκB activation and downstream neuroinflammation^69^. Overall, these data highlight miR-451a as a key regulator of synaptic integrity and a potential target for therapies addressing both cognitive and depressive symptoms.

The upregulation of miR-151a-5p and miR-4516, along with the downregulation of let-7b-3p and miR-100-5p following INCREM treatment points toward additional regulatory pathways. Although less studied in MDD, miR-4516 levels are reduced in CSF extracellular vesicles and in dlPFC of individuals with MDD who died by suicide^43,70,71^. The link between miR-4516 and suicide is mainly based on its predicted regulation of the *SLC6A4* gene (which encodes the SERT protein) and other genes involved in cell adhesion and synaptic signaling (e.g., *NR3C1* which encodes the glucocorticoid receptor)^70,72^. Similarly, miR-151a-5p is reduced in circulating extracellular vesicles from adolescents with anxiety disorders^73^, while miR-151-3p (originating from the same precursor gene hsa-mir-151a) is modulated by SSRI paroxetine in human lymphoblastic cell lines derived from healthy adult females^58^. The let-7 family is involved in neurodegeneration, cell survival, and synaptic development^74^. Recently, we reported that this miRNA family is consistently downregulated by lithium treatment in both responder or non-responder groups of patients with bipolar disorder^75^, and elevated levels have been found in the plasma of patients with substance use disorders, which share overlapping neural circuits with MDD^76,77^. While miR-100-5p is known as a tumor suppressor and its involvement in vital cellular processes through the regulation of targets such as mTOR, FOXO1, AGO2 mRNAs, among others^78,79^, its role in MDD remains unclear. Recently, it has been linked to shared gene networks between amyotrophic lateral sclerosis and depression^80^. Overall, this four-miRNA profile, though requiring validation, points to the recruitment of master regulators involving synaptic maintenance and stress adaptation, providing further molecular context for the broad therapeutic effects of INCREM.

These exploratory hypotheses are further supported by the functional enrichment analysis, which revealed that the INCREM-specific miRNAs target a potential pro-cognitive biological network. The identified enriched pathways, which include neurotrophin signaling, axonal guidance, long-term potentiation (LTP), and glutamatergic and GABAergic synaptic transmission, as well as the cognitive pathway itself, are known to contribute to learning and memory^81,82^. Cognitive remediation is fundamentally a form of structured, guided learning designed to recruit and strengthen compromised neural circuits, mainly in the prefrontal and parietal networks^83–85^. The findings of this study suggest a potential molecular correlate for these processes. INCREM therapy may act as an environmental stimulus, inducing changes in miRNA expression that could influence genes involved in synaptic strengthening (LTP), structural remodeling (axon guidance), and rebalancing of the excitatory/inhibitory tone. Therefore, it could be hypothesized that INCREM improves cognitive and functional performance in MDD by modulating a specific set of circulating miRNAs that regulate gene networks implicated in brain plasticity.

In contrast, the molecular trajectory of PSYCHOED (upregulation of miR-126-5p and miR-195-5p) suggests an alternative exploratory hypothesis based on systemic adaptation rather than direct synaptic remodeling. A growing body of literature demonstrates that miR-195-5p is a potent negative regulator of the Wnt/β-catenin signaling pathway^86,87^. This pathway is a master regulator of cell fate, including proliferation, differentiation, and apoptosis, and its dysregulation is implicated in numerous diseases with inflammatory components^88,89^. However, there are few studies on the role of miR-195-5p in MDD. Changes in plasma miR-195-5p levels have recently been reported in patients with treatment-resistant depression following ECT, although the effect was not sustained^90^. A preclinical study reported that, in rats exposed to chronic stress, miR-195-5p expression levels decreased in the mPFC and increased in the ventral tegmental area, with stronger effects in resilient versus anhedonic rats^91^. The role of miR-126-5p in MDD remains unclear, though recent findings showed altered expression in plasma extracellular vesicles of adolescents with MDD compared to healthy peers^92^. However, miR-126-5p has been mainly involved in multiple sclerosis with high psychiatric comorbidity, being a key regulator of vascular health and neuroinflammation. Its dysregulation (either too high or too low) appears to be a common mechanism in neurological disorders involving blood-brain barrier dysfunction and oxidative stress^93,94^.

The functional enrichment analysis reinforces this distinction. Unlike INCREM, the PSYCHOED targetome showed no enrichment in cognitive and synaptic pathways. Instead, it converges on pathways involved in cell fate and intercellular communication, such as cell senescence, Wnt signaling, cell cycle, and Hippo signaling. This distinct profile suggests that PSYCHOED may influence different biological processes to those associated with INCREM, opening up new hypotheses on how supportive therapies exert their effects. MDD is increasingly recognized as a systemic disorder associated with chronic inflammation, oxidative stress, and accelerated biological aging^95–97^. By providing patients with disease management tools and reducing psychological distress, psychoeducation may act as a systemic buffer against the pathological consequences of chronic stress. It is therefore biologically plausible that the benefits of PSYCHOED, manifested as improved daily functioning (FAST) without objective cognitive gains, derive from improved systemic cellular resilience rather than specific synaptic circuit remodeling. Modulation of the Wnt pathway is consistent with this interpretation of a broad, systemic effect on cellular integrity. Thus, “targeted” interventions (e.g. INCREM) may function by modulating cognitive circuits and their plasticity, while “supportive” therapies (e.g. PSYCHOED) act through broader effects on systemic resilience to stress.

### Translational implications and study limitations

The findings of this exploratory study have direct translational implications, addressing the critical need for objective biomarkers in psychiatry^98^. The stability and accessibility of circulating miRNAs make them ideal candidates for liquid biopsy approaches to monitor therapeutic target engagement. However, we acknowledge several limitations, primarily the small size and the imbalance between study arms. The unequal sample sizes across study arms resulted from differential dropout rates. Although participants were initially assigned using block randomization to ensure balanced groups, attrition was higher in the PSYCHOED condition and could not be mitigated. This imbalance may also suggest greater participant engagement or acceptability of the INCREM intervention compared to the control (PSYCHOED) condition, which should be considered when interpreting the results, as the influence of a variety of factors cannot be entirely ruled out in a sample of this size. We addressed these shortcomings by using an intra-subject longitudinal design with rigorous statistical correction (Šidák adjustment), implemented some control measures to account for the effects of antidepressant medication (not allowing changes prior and during the study period). Furthermore, the identification of large-effect sizes reinforces the preliminary validity of these exploratory findings, despite the restricted sample. While clinical follow-up data were available, miRNA measurements were limited to pre- and post-intervention time-points, preventing longitudinal molecular analyses. Although plasma miRNAs may reflect CNS processes, their cellular origin is heterogeneous; future studies using neuron-derived extracellular vesicles could provide greater specificity regarding brain-related changes^36,38^. MiRNA signatures showed an association with response, but the correlational nature of this study limits definitive causal interpretations. Due to the small sample size, reliable correlation analyses between changes in miRNA expression and functional and cognitive outcomes could not be performed. Future directions should focus on validating these findings in multicenter and diverse cohorts. This study focused on a specific panel of miRNAs; however, other small non-coding RNAs, such as transfer RNA fragments (tRFs), may also contribute to MDD pathophysiology and should be explored using high-throughput sequencing. Importantly, we must emphasize that the biological interpretations presented here—particularly those derived from in silico target prediction and pathway enrichment—must be considered exploratory hypotheses rather than definitive mechanistic conclusions. Direct experimental validation (e.g., luciferase reporter assays or in vitro functional studies) is essential to confirm the functional significance of these miRNA-target interactions. Finally, integrating miRNA profiling with other modalities, such as functional neuroimaging, will allow changes in circulating biomarkers to be directly linked to the activity and connectivity of the brain circuits involved.

## Conclusions

This study represents a significant conceptual advance, moving the understanding of psychological interventions from a purely behavioral framework to one with a measurable biological readout. INCREM appears to directly modulate gene networks governing neuroplasticity and synaptic function, which mirrors objective cognitive recovery, while PSYCHOED engages pathways related to systemic cellular resilience and homeostasis. Although replication in larger cohorts is essential, the identified circulating miRNA profiles offer a promising foundation for the development of clinically actionable biomarkers for response monitoring and treatment assignment. Specifically, these signatures could serve as a liquid biopsy to assess therapeutic target engagement or to guide precision psychiatry by personalizing intervention selection based on the patient’s baseline molecular profile. Ultimately, this research paves the way for a framework where psychological interventions are optimized and monitored through objective biological indicators in patients suffering from cognitive dysfunction in MDD.

## Supplementary Information

Below is the link to the electronic supplementary material.

Supplementary Material 1

## Data Availability

The data that support the findings of this study are available from the corresponding authors, AB, upon reasonable request.
