# Supplementary material for: Peripheral miRNA profiling identifies therapy-specific biological trajectories in the treatment of cognitive dysfunction in depression: an exploratory mechanistic study
Source: Sci Rep. 2026 May 29;16:24649. doi: 10.1038/s41598-026-55039-1 (PMC13454330; doi:10.1038/s41598-026-55039-1)
Supplement: Supplementary file 1 [file 41598_2026_55039_MOESM1_ESM.pdf]

Supplemental Material

Peripheral miRNA profiling identifies therapy-specific biological trajectories in the treatment of cognitive dysfunction in depression: An exploratory mechanistic study

Lluis Miquel-Rio[1,2,3,#], Muriel Vicent-Gil[3,4,5,6,#], Judith Jericó-Escolar[1,2,3,7], Júlia Carrasco-Hernández[4], Miriam Jubero[4], Verónica Paz[1,2,3], Leonor Gawron[8], Esther Ruiz-Bronchal[1,2,3], Javi Vera[5], Caterina del Mar Bonnín[5], Dolors Puigdemont[3,4,5], Carlo Alemany[3,4,5], Narcís Cardoner[3,4,5,6], Javier de Diego Adeliño[3,4,5,6], Analia Bortolozzi[1,2,3*], and Maria J Portella[3,4,5,6,*]

[1]Institute of Biomedical Research of Barcelona (IIBB), Spanish National Research Council (CSIC), 08036 Barcelona, Spain

[2]Systems Neuropharmacology Research Group, August Pi i Sunyer Biomedical Research Institute (IDIBAPS), 08036 Barcelona, Spain

[3]Biomedical Research Networking Center for Mental Health (CIBERSAM), Institute of Health Carlos III (ISCIII), 28029 Madrid, Spain

[4]Sant Pau Mental Health Group, Institut de Recerca Sant Pau (IR SANT PAU), Sant Quintí 77-79, 08041 Barcelona, Spain

[5]Hospital de la Sant Creu i Sant Pau, Sant Antoni Maria Claret 167, 08025 Barcelona, Spain

[6]Department of Psychiatry and Legal Medicine, Universitat Autònoma de Barcelona (UAB), 08193 Bellaterra, Spain.

[7]Faculty of Medicine and Health Sciences, University of Barcelona (UB), 08036 Barcelona, Spain

[8]Addictions Unit, Psychiatry and Psychology Service, ICN, Hospital Clinic Barcelona; Health and Addictions Research Group, IDIBAPS, 08036 Barcelona, Spain

#Contributed equally to the manuscript as the first authors

*Correspondence: analia.bortolozzi@iibb.csic.es or mportella@santpau.cat

Supplementary Table 1. List of miRNA primers used in the study

Primer Forward ID	Primer Forward SEQ	Primer Reverse SEQ
**let-7b-3p Forward**	TACAACCTACTGCCTTCCCAAA	ACCTCAGAGTCACTATGGCACT
**let-7b-5p Forward**	GCCGTAGTAGGTTGTGTGGTTA	ACCTCAGAGTCACTATGGCACT
**miR-100-5p Forward**	CCGTAGATCCGAACTTGTGAAA	ACCTCAGAGTCACTATGGCACT
**miR-126-5p Forward**	CCGCTTATTACTTTTGGTACGC	ACCTCAGAGTCACTATGGCACT
**miR-129-5p Forward**	TTTGCGGTCTGGGCTTGC	ACCTCAGAGTCACTATGGCACT
**miR-1299 Forward**	TTCTGGAATCCTGTGTGAGGGA	CTCAGAGTCACTATGACACT
**miR-135a-5p Forward**	GCTGGGTATGGCTTTTTATTCCTA	ACCTCAGAGTCACTATGGCACT
**miR-144-3p Forward**	GCTACAGTATAGATGATGTACTAA	CTCAGAGTCACTATGACACT
**miR-151a-3p Forward**	AGACTGAAGCTCCTTGAGGAAA	CTCAGAGTCACTATGACACT
**miR-151a-5p Forward**	CGGAGCTCACAGTCTAGTAAA	ACCTCAGAGTCACTATGGCACT
**miR-151b Forward**	CCGAGGAGCTCACAGTCTAAA	ACCTCAGAGTCACTATGGCACT
**miR-181a-3p Forward**	GGCGACCGTTGATTGTACC	ACCTCAGAGTCACTATGGCACT
**miR-187-3p Forward**	GCTCGTGTCTTGTGTTGCAG	ACCTCAGAGTCACTATGGCACT
**miR-195-5p Forward**	TAGCAGCACAGAAATATTGGCAA	ACCTCAGAGTCACTATGGCACT
**miR-199a-3p Forward**	ACAGTAGTCTGGACATTGGTTA	CTCAGAGTCACTATGACACT
**miR-199a-5p Forward**	GGTGTTCAGACTACCTGTTCAA	ACCTCAGAGTCACTATGGCACT
**miR-21-5p Forward**	CGGCTTATCAGACTGATGTTGA	ACCTCAGAGTCACTATGGCACT
**miR-219a-2-3p Forward**	ATTGTGGCTGGACATCTGTAAA	ACCTCAGAGTCACTATGGCACT
**miR-219a-5p Forward**	GATTGTCCAAACGCAATTCTAAA	CTACGACCTCAGAGTCACTATG
**miR-23b-3p Forward**	CATTGCCAGGGATTACCACAAA	CTACGACCTCAGAGTCACTATG
**miR-27b-3p Forward**	GACACAGTGGCTAAGTTCTGC	ACCTCAGAGTCACTATGGCACT
**miR-27b-5p Forward**	GCTTAGCTGATTGGTGAACAA	ACCTCAGAGTCACTATGGCACT
**miR-320c Forward**	GGCTGGGTTGAGAGGGTAAA	ACCTCAGAGTCACTATGGCACT
**miR-335-5p Forward**	CAAGAGCAATAACGAAAAATGTA	CTACGACCTCAGAGTCACTATG
**miR-338-5p Forward**	CCAGCATCAGTGATTTTGTTG	CTACGACCTCAGAGTCACTATG
**miR-33a-3p Forward**	AATGTTTCCACAGTGCATCACAA	CTACGACCTCAGAGTCACTATG
**miR-34c-3p Forward**	AGGCAGTGTAGTTAGCTGATTG	CTACGACCTCAGAGTCACTATG
**miR-378a-3p Forward**	CACTTGGAGTCAGAAGGCAAA	ACCTCAGAGTCACTATGGCACT
**miR-423a-5p Forward**	AGGGGCAGAGAGCGAGACTTTA	ACCTCAGAGTCACTATGGCACT
**miR-4516 Forward**	AGAAGGGTCGGGGCAAA	ACCTCAGAGTCACTATGGCACT
**miR-451a Forward**	ACCGTTACCATTACTGAGTTA	CTACGACCTCAGAGTCACTATG
**miR-483-5p Forward**	ACGGGAGGAAAGAAGGGAGAAA	CTCAGAGTCACTATGACACT
**miR-499a-5p Forward**	ACGAGACTTGCAGTGATGTTTA	ACCTCAGAGTCACTATGGCACT
**miR-5584-5p Forward**	CAGGGAAATGGGAAGAACTAGAA	CTACGACCTCAGAGTCACTATG
**miR-574-5p Forward**	ACGCTCATGCACACACCCACAAA	ACCTCAGAGTCACTATGGCACT
**miR-625-5p Forward**	CTGGGGAAAGTTCTATAGTCCA	ACCTCAGAGTCACTATGGCACT
**miR-642a-5p Forward**	GCCCTCTCCAAATGTGTCTTGAA	ACCTCAGAGTCACTATGGCACT
**miR-6734-5p Forward**	GAGGGGAGAATGAGGTGGAGAAA	CTCAGAGTCACTATGACACT

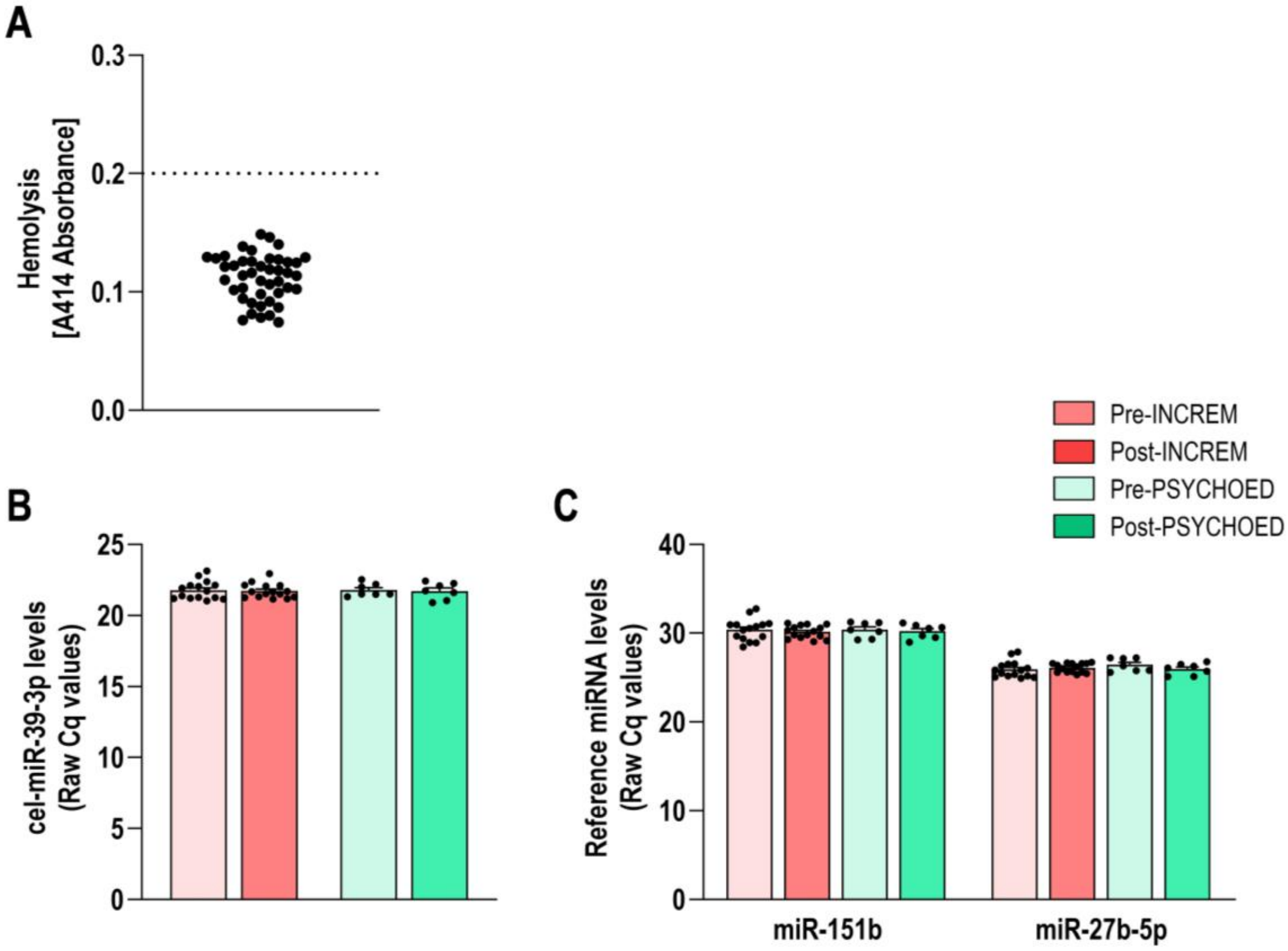

Supplementary Figure 1. Quality control of plasma samples. (A) Plasma hemolysis was assessed by measuring free hemoglobin absorbance at 414 nm (NanoDrop™). All samples passed the exclusion threshold A414 nm < 0.2. **(B)** Technical efficiency of RNA purification and quantification was confirmed by the stable Cq values of the exogenous spike-in (cel-miR-39-3p) across all samples. **(C)** The NormFinder algorithm identified hsa-miR-151b and hsa-miR-27b-5p as the most stable reference pair (stability value = 0.074). This stability was validated by a three-way ANOVA on raw Cq values, which showed no significant effects ($p > 0.05$) across experimental conditions (detailed statistical analysis in Supplementary Excel File 2).

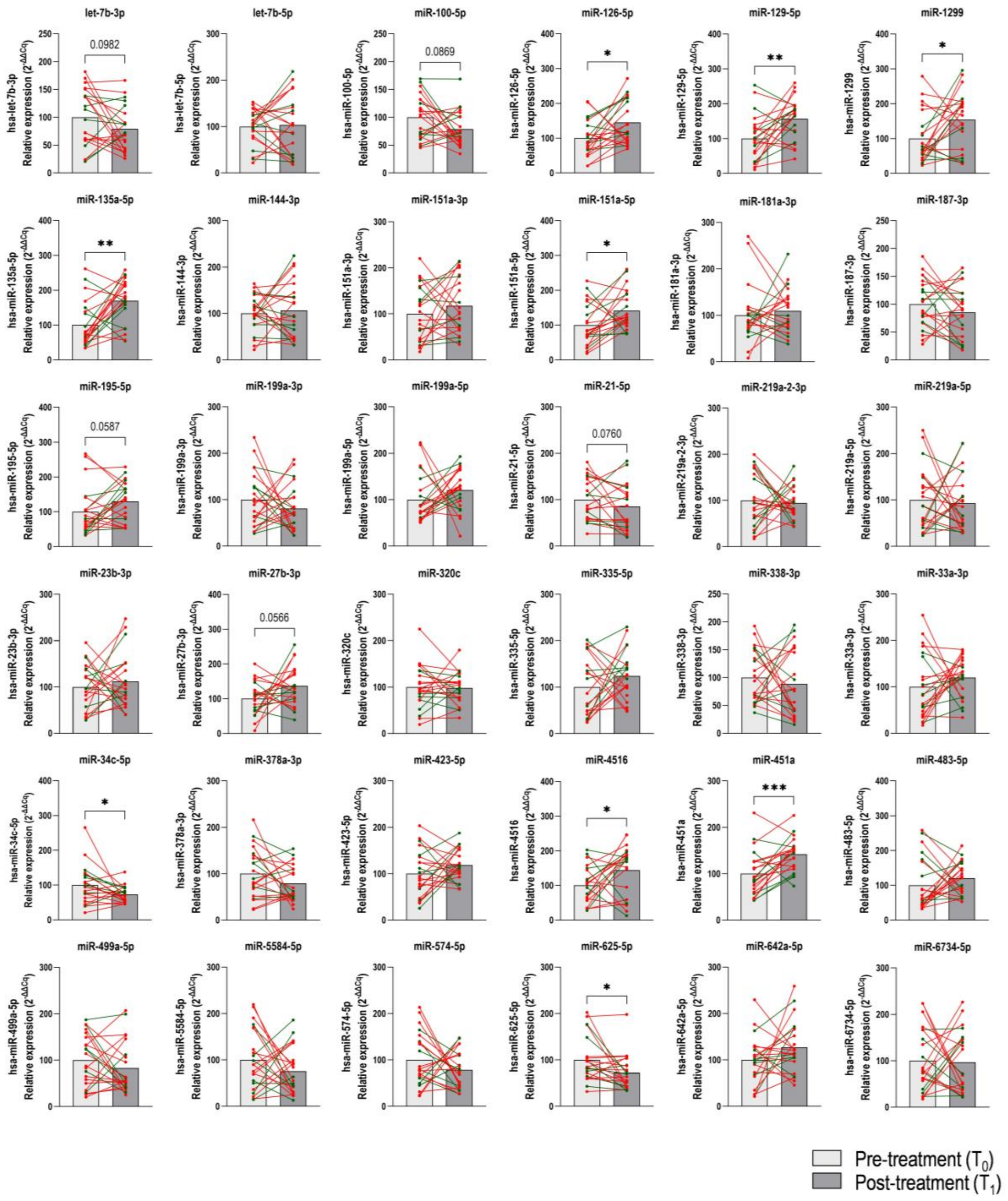

Supplementary Figure 2. Plasma miRNA levels in MDD patients following psychological intervention (INCREM and PSYCHOED). Analysis of a panel of 36 miRNAs in plasma samples taken from MDD patients before (pre-treatment) and after (post-treatment) undergoing integral cognitive remediation (INCREM, in red) or psychoeducation (PSYCHOED, in green). Significant increases were detected in seven miRNAs: miR-126-5p, miR-129-5p, miR-1299, miR-135a-5p, miR-151a-5p, miR-4516 and, miR-451a, and significant decreases in two miRNAs: miR-34c-5p and miR-625-5p when comparing pre- and post-treatment values. Data are expressed as mean ± SEM. *$p < 0.05$, **$p < 0.01$, ***$p < 0.001$ when comparing the values before and after treatment (detailed statistical analysis in Supplementary Excel File 2).

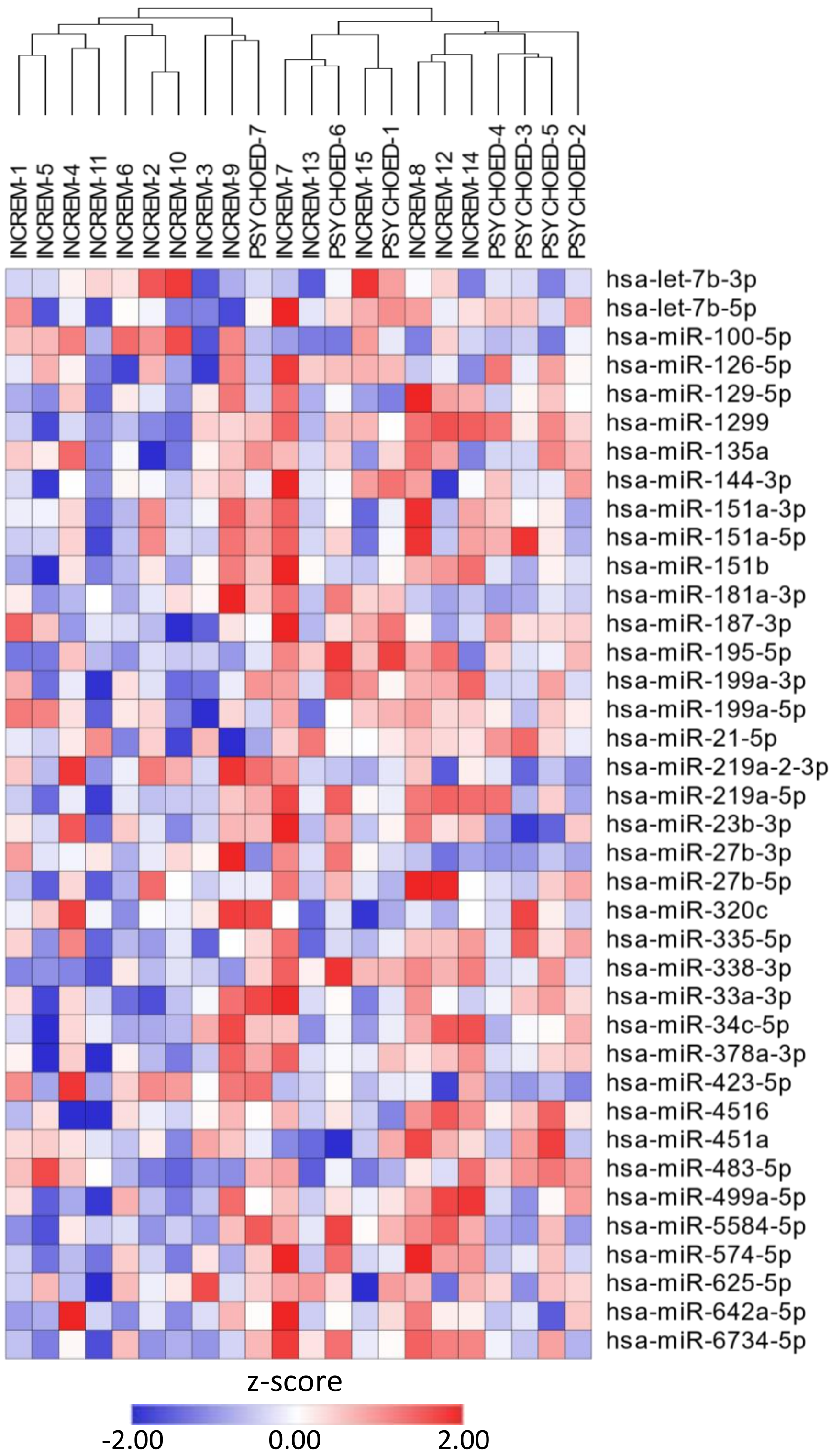

Supplementary Figure 3. Hierarchical clustering shows the expression of 36 miRNAs among INCREM- and PSYCHOED-treated patients with MDD. The intensity of the color represents the expression status. Red represents higher expression and blue represents reduced expression.

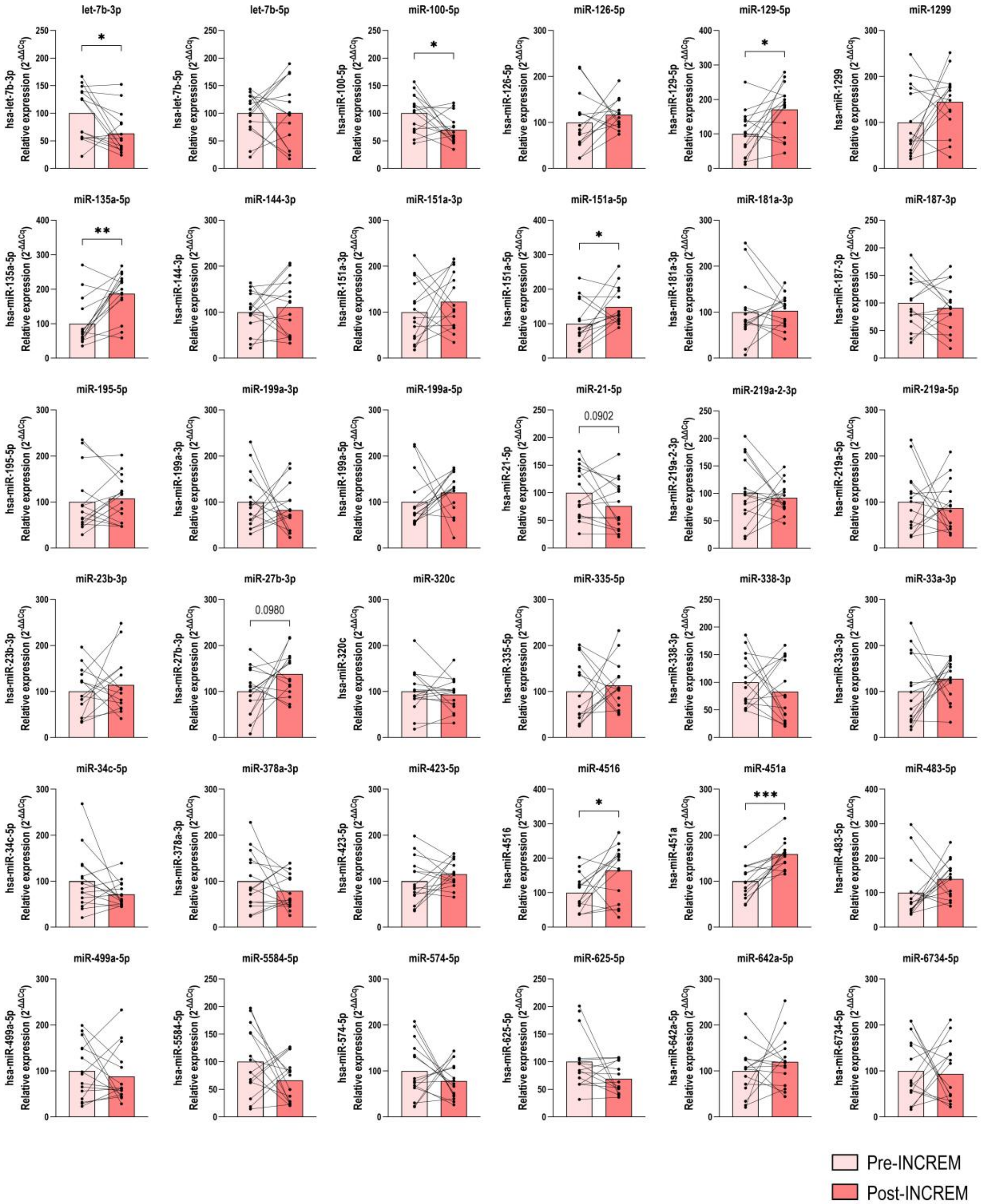

Supplementary Figure 4. Plasma miRNA levels in MDD patients following INCREM intervention. Analysis of a panel of 36 miRNAs in plasma samples taken from MDD patients before (pre-treatment) and after (post-treatment) undergoing integral cognitive remediation (INCREM). Significant increases were detected in five miRNAs: miR-129-5p, miR-135a-5p, miR-151a-5p, miR-4516 and, miR-451a, and significant decreases in let-7b-3p and miR-100-5p when comparing pre- and post-treatment values. Data are expressed as mean ± SEM. $*p < 0.05$, $**p < 0.01$, $***p < 0.001$ when comparing the values before and after treatment (detailed statistical analysis in Supplementary Excel File 2).

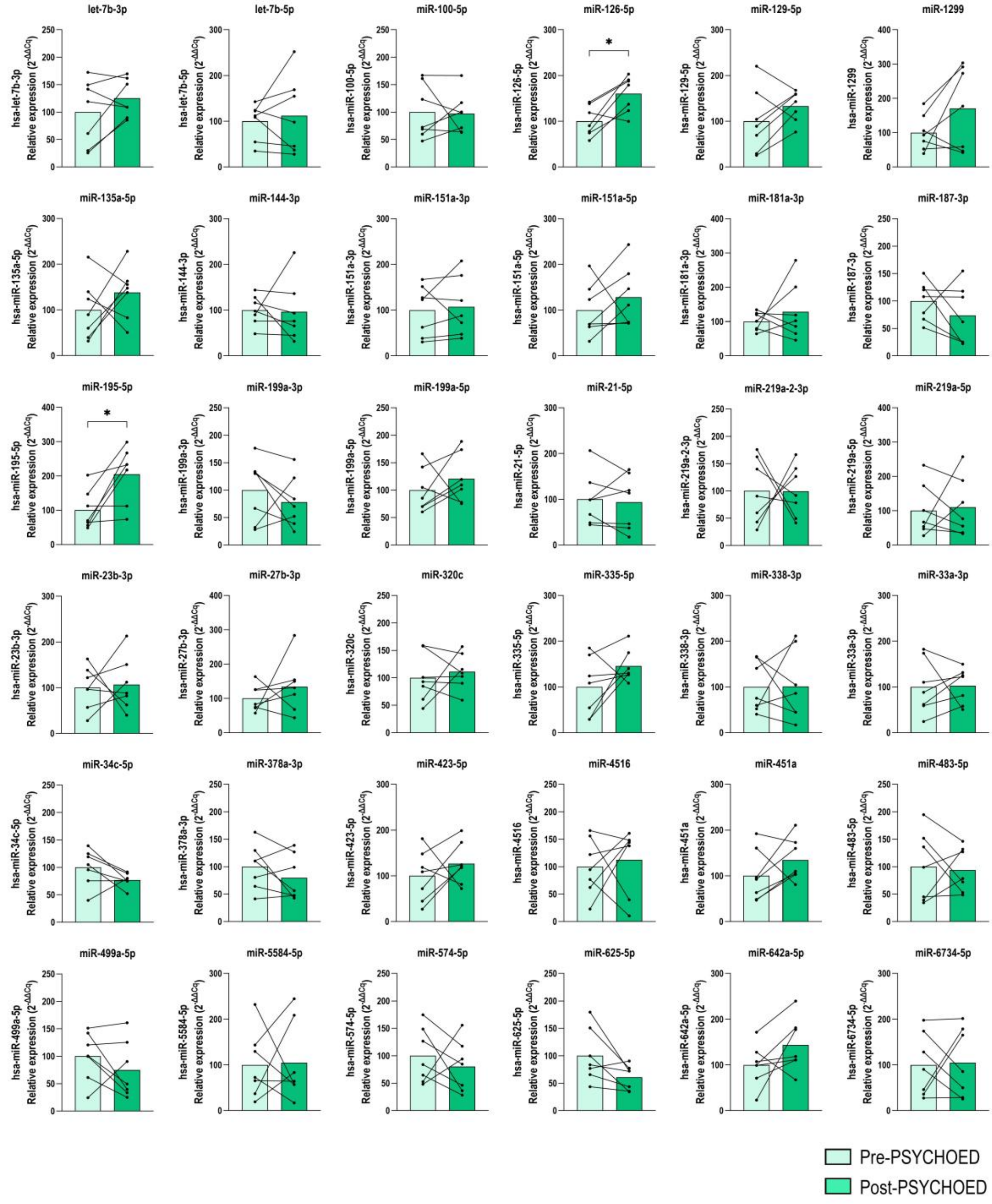

Supplementary Figure 5. Plasma miRNA levels in MDD patients following PSYCHOED intervention. Analysis of a panel of 36 miRNAs in plasma samples taken from MDD patients before (pre-treatment) and after (post-treatment) undergoing psychoeducation (PSYCHOED). Significant increases were detected in miR-126-5p and miR-195-5p when comparing pre- and post-treatment values. Data are expressed as mean ± SEM. *$p < 0.05$ when comparing the values before and after treatment (detailed statistical analysis in Supplementary Excel File 2).

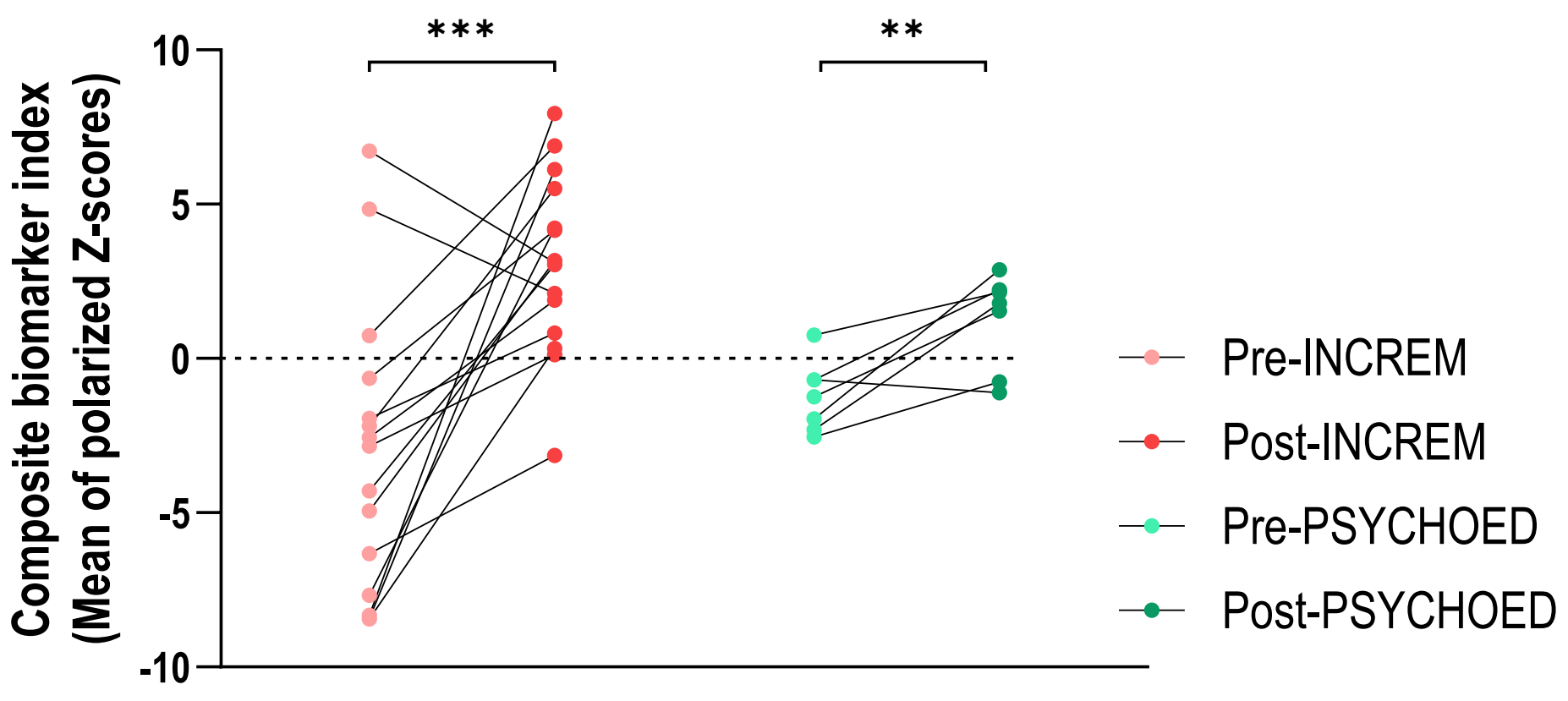

Supplementary Figure 6. Longitudinal changes in the composite miRNA biomarker index following treatment. Individual trajectories of the composite biomarker index for the INCREM (red, n=15) and PSYCHOED (green, n=7) groups. The index was calculated as the mean of polarized Z-scores for the identified treatment-responsive miRNAs in each group (7 miRNAs for INCREM; 2 miRNAs for PSYCHOED). Data points represent individual subjects at pre- and post-treatment time points. Statistical significance was determined by paired t-tests. **p < 0.01**, ***p < 0.001 when comparing the values before and after treatment (detailed statistical analysis in Supplementary Excel File 2).
